# Growth mechanism of metal-oxide nanowires synthesized by electron beam evaporation: A self-catalytic vapor-liquid-solid process

**DOI:** 10.1038/srep06589

**Published:** 2014-10-10

**Authors:** Hak Ki Yu, Jong-Lam Lee

**Affiliations:** 1 Division of Advanced Materials Science and Department of Materials Science and Engineering, Pohang University of Science and Technology (POSTECH), Pohang, 790-784, Korea; 2 Current address: Max-Planck-Institut Für Biophysikalische Chemie, am Fassberg 11, 37077 Göttingen, Germany.

## Abstract

We report the growth mechanism of metal oxide nanostructures synthesized by electron beam evaporation. The condensed electron beam can easily decompose metal oxide sources that have a high melting point, thereby creating a self-catalytic metal nanodot for the vapor-liquid-solid process. The metal oxide nanostructures can be grown at a temperature just above the melting point of the self-catalyst by dissolving oxygen. The morphology of nanostructures, such as density and uniformity, strongly depends on the surface energy and surface migration energy of the substrate. The density of the self-catalytic metal nanodots increased with decreasing surface energies of the substrate due to the perfect wetting phenomenon of the catalytic materials on the high surface energy substrate. However, the surfaces with extremely low surface energy had difficulty producing the high density of self-catalyst nanodot, due to positive line tension, which increases the contact angle to >180°. Moreover, substrates with low surface migration energy, such as single layer graphene, make nanodots agglomerate to produce a less-uniform distribution compared to those produced on multi-layer graphene with high surface migration energy.

One-dimensional metal oxide nanostructures have great potential in a huge number of application fields, including energy and environmental science, electronics, photonics, and biology^1^,^2^,^3^,^4^,^5^. The growth mechanism of the metal oxide nanostructures can mainly be divided into two categories, based on whether or not metallic catalysts are used^6^,^7^,^8^. The vapor-liquid-solid (VLS) growth mode uses nano-sized metal dots (e.g., Au, Ni) at the early stage of growth and the immersed supersaturated atoms during growth, segregated as the metal oxide nanostructures near the melting point of the catalyst metal. On the other hand, the vapor-solid (VS) growth mode is strongly affected by the anisotropic properties between crystal orientations of nano-sized oxide seed particles, resulting in the facile synthesis of nanostructures. Compared to the VS mode, the VLS mode has the advantages of i) freedom of oxide material selection, ii) a simple process for the nanostructure growth at temperature just above the melting point of the metal catalyst.

A drawback of the VLS process is that, the metal catalyst can react with the target materials during growth at high temperatures, thereby creating intermetallic compounds and contamination^9^,^10^. Therefore, VLS that uses the metal element of the target metal oxide as a catalyst (i.e., self-catalyst) has been attempted by decomposing the metal oxide during growth for formation of high quality products without contamination^11^,^12^,^13^,^14^. However, the decomposition temperature of most metal oxides is too high to control. Carbo-thermal decomposition, in which graphite powder is mixed with the metal oxide powder, can be used to reduce the decomposition temperature, but this process also has contamination problems, such as metal carbide formation^13^,^14^. Alternatively, use of an electron beam can decompose several materials that have a high melting point. Several groups have attempted electron beam evaporation to grow nanowires such as Si, Ge, and SnO_2_^15^,^16^,^17^. In most of the studies, an Au nanodot was used as the catalyst during VLS. Catalysts that have low melting points, such as In and Bi, have been used to reduce the growth temperatures, but they are heterogeneous catalysts^18^,^19^. For the case of metal oxides, heterogeneous catalysts are not needed if the metal element of the metal oxide can be used by decomposing it during growth. Moreover, the decomposition temperature of metal oxide can be reduced under conditions of low oxygen chemical potential, such as in a high vacuum^20^. Most metal oxides can be easily decomposed by the condensed electron-beam, and sufficient metal flux for the self-catalyst of the VLS process can be formed under a low oxygen partial pressure. In this case, the VLS growth can be achieved easily, at a temperature just above the melting point of the metallic self-catalyst, as shown in Fig. 1. The electron beam can easily achieve the vapor pressure required for self-catalyst formation, compared to the conventional tools for nano-structure growth, such as a heat furnace and pulsed laser. Because the maximum temperature of a normal heat furnace is <1,500°C, it has limited application for metal oxides with decomposition temperatures > 1,500°C. Moreover, the pulsed laser tool tends to form the stoichiometrically identical composition to the target source material during laser irradiation, without decomposing of the metal oxide. Overall, the electron beam evaporation is the most ideal tool for nanowire growth using self-catalysis by decomposing of the metal oxide during irradiation.

In this work, we focused on the group 13 elements in the periodic table (Al, Ga, and In) that have high decomposition temperatures for their metal oxides, but, low melting points of metals themselves, as shown in Fig. 2a (Refer to supplementary information S1 for the growth of MgO nanowire by electron beam evaporation using MgO pellet as a source material without use of a catalyst). By checking the morphological changes at temperature below and above the melting point of the metal, self-catalytic VLS of electron beam-evaporated group 13 oxides can be verified. To learn the growth mechanism of liquid metal mediated VLS growth for the metal oxide nanostructures, the effect of surface energy on the nucleation of the self-catalytic nanodots was examined. Although high surface energy is helpful for conventional heterogeneous nucleation, it does not form a nano-sized droplet, but tends to spread easily, resulting in low density of nanostructures^21^. Finally, the effect of surface migration energy on the nucleation of the self-catalytic nanodots for the VLS process was studied using an n-graphene layer as the substrate, because the surface energy of n-graphene is independent of n, whereas the surface migration energy is strongly dependent on it^22^,^23^,^24^.

## Results and Discussion

**Figure 2:**
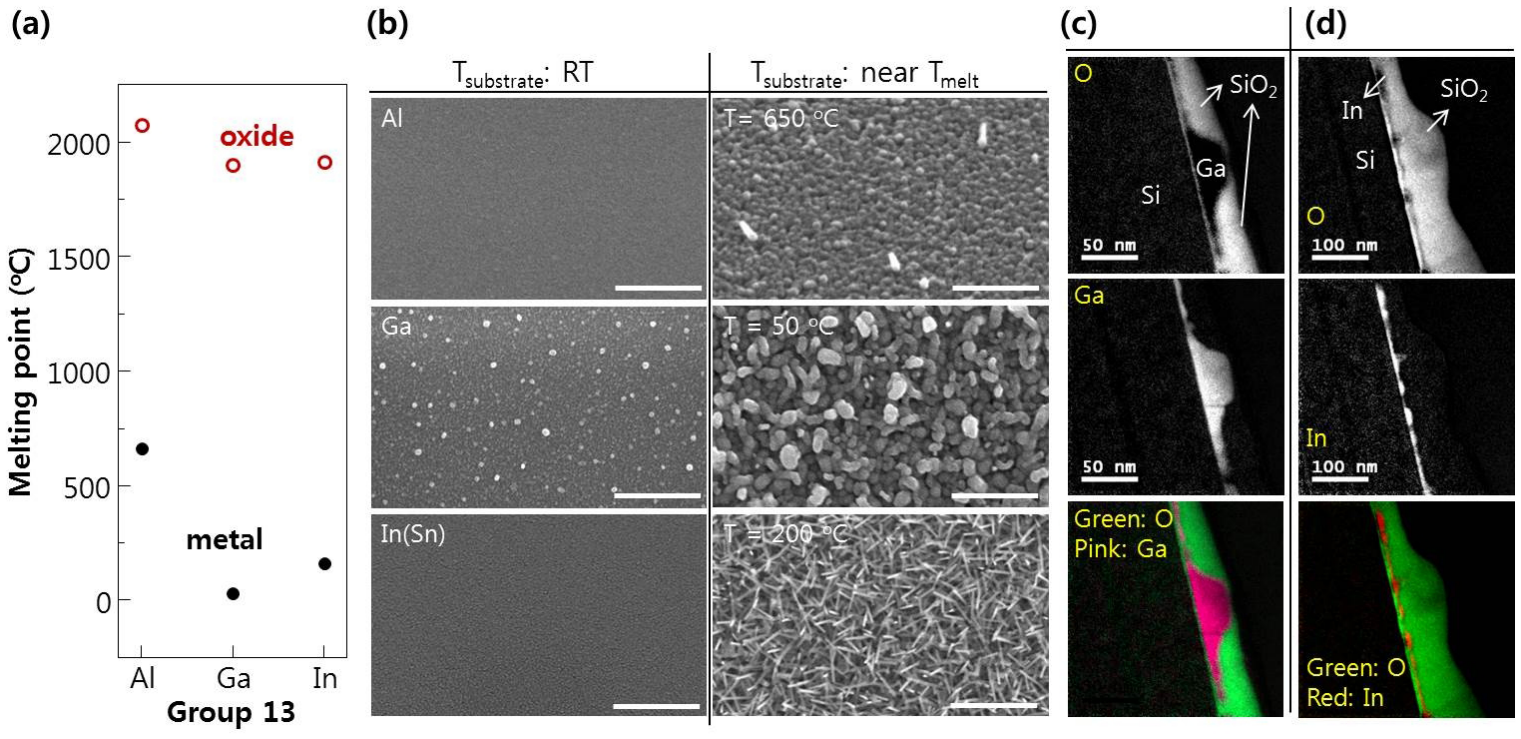
(a) Melting point of group 13 elements (pure metal and metal oxide). (b) Top view SEM images of electron beam-evaporated group 13 oxide materials on SiO_2_ covered Si (100) wafer at RT, and near the melting point of the pure 13 group metals. Scale bar, 500 nm. TEM-EELS maps (oxygen, metal element: gallium and indium) and color contour maps of electron beam evaporated (c) gallium oxide and (d) indium(tin) oxide on Si (100) substrate at the early stage of growth.

SEM images (Fig. 2b) were taken of electron beam-evaporated group 13 oxide materials on SiO_2_ covered Si (100) wafers at room temperature (RT), and near melting point of the pure metals (Al: 660.03°C, Ga: 29.77°C, and In: 156.6°C). The surface morphologies of the samples were quite flat at RT, except for Ga_2_O_3_, whereas there were several nanostructures near the melting point of the pure metal. The rough surface of Ga_2_O_3_ at RT resulted from the low melting point of pure Ga, and the even lower melting point of the nano-sized dot than the bulk state; as a result, the liquid phase occurred during deposition even at RT^25^,^26^. These metal oxides could be decomposed easily by a high density electron-beam, and sufficient metal flux for self-catalysis of the VLS process can be formed under a low oxygen partial pressure. In this case, VLS growth can be achieved easily at a temperature just above the melting point of the metallic self-catalyst, resulting in development of nanostructures. To verify that self-catalytic VLS growth occurs, the most important is the existence of metallic nanodots at the early stages of growth.

Figure 2c and 2d show the electron energy loss spectroscopy in a transmission electron microscope (TEM-EELS) maps of electron beam-evaporated gallium oxide and indium(tin) oxide (ITO) exposed for just for 10 sec on Si (100) substrate at 100°C for gallium oxide, and 300°C for indium(tin) oxide. To protect against oxidation during transfer from the electron beam evaporator to the TEM, each sample was passivated *in-situ* using an amorphous SiO_2_ layer (~70 nm thick). In the maps, oxygen and the metal element were separated by a clear line, which clearly proves the existence of metallic nanodots at the initial stage of nanostructure growth. Based on this clue about metallic nanodot formation, we can study the growth mechanism of these nanodots on various substrates, and the evolution of their final nanostructure.

Hereafter, we focused on the ITO nanostructure, because it showed a relatively uniform structure due to the moderate melting point of In compared to those of Al and Ga. Moreover, several recent studies about the ITO nanowires reflect the importance of not only applications based on excellent metallic conductivity with the transparency but also growth mechanism itself^27^,^28^,^29^. Studies of ITO nanowire growth with Au or Au-Cu nanodots as catalysts clearly show the role of Sn during the VLS process^27^,^28^. However, the growth behavior will differ when a self-catalyst is used, because the atomic diffusion and reactivity of In and Sn in Au or Au-Cu matrix will be different from those in the pure self-catalyst. Furthermore, a previous study of self-catalytic VLS growth of ITO nanowires focused on several growth factors such as incident flux angle, growth rate, and substrate temperature^29^. Study of the early stage of growth on various substrates expand understanding of self-catalytic VLS growth.

**Figure 3:**
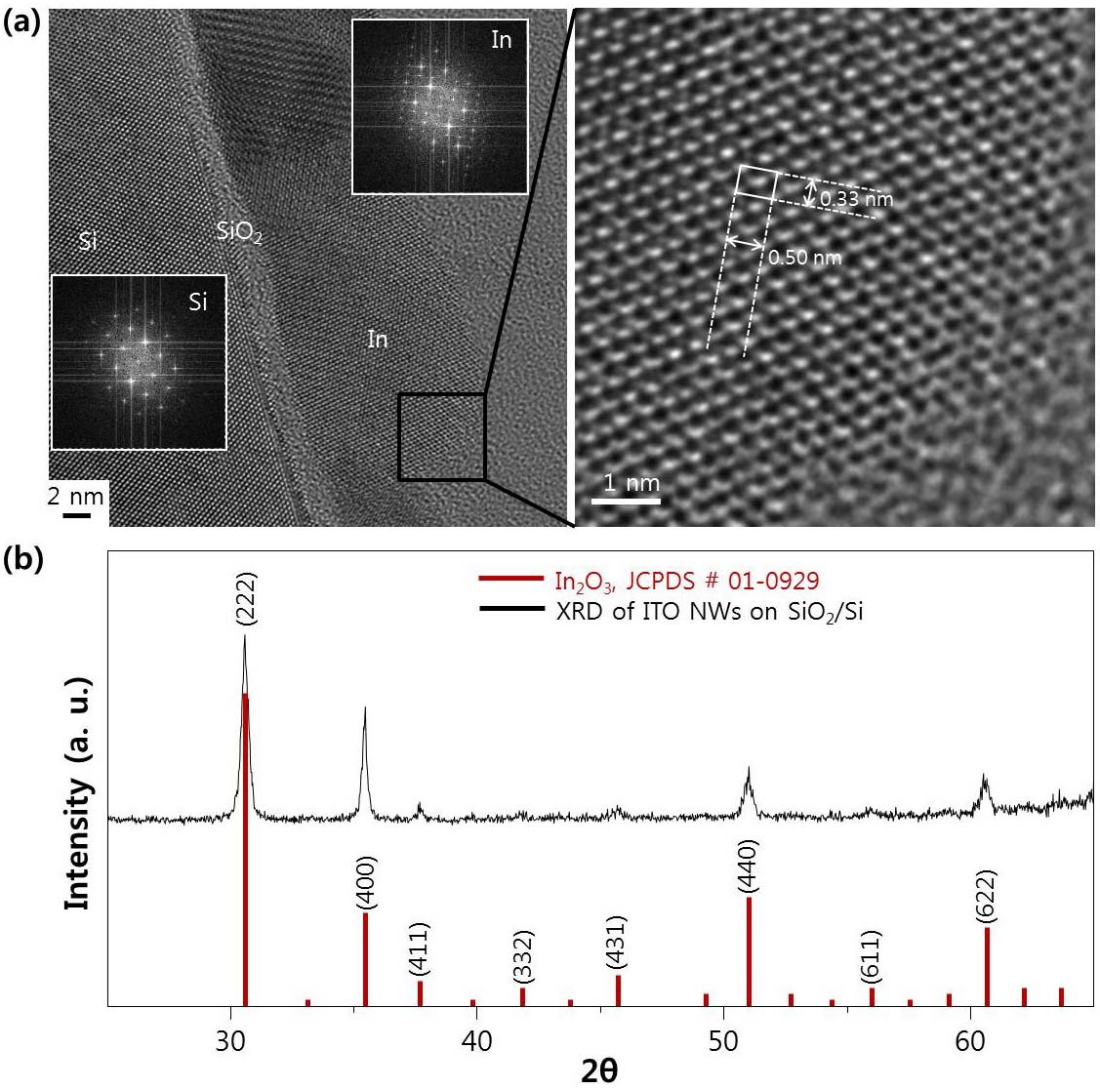
(a) HR-TEM image of metallic indium head at the early stage of growth. (b) XRD result of In(Sn) oxide nanowires grown on SiO_2_/Si(100) during 150 sec at 300°C (with JCPDS profile of In_2_O_3_).

Above all, we studied the composition of the ITO nanowire (metallic head with oxide body) to determine its growth mechanism. The existence of a metallic nanodot head for VLS growth was confirmed by high resolution TEM (HR-TEM). The TEM sample was prepared by evaporating the ITO pellet in an e-beam system for 10 s at a substrate temperature of 300°C on SiO_2_/Si (100). The well-crystallized tetragonal metallic In phase (a = b = 0.33 nm and c = 0.50 nm) was observed clearly (Fig. 3a). Sn was also detected (Supplementary Fig. S3). The metallic In nanodots were 20 nm in diameter; they were formed by decomposition of the oxide source during electron beam irradiation, resulting in the VLS process for nanowire growth. To determine the exact composition of the oxide body, X-ray diffraction was measured (Fig. 3b). The peak positions of the ITO nanowires were identical to the JCPDS profile of In_2_O_3_^30^. During nanowire growth from the self-catalytic metal head by segregation of the supersaturated oxygen atoms, the most thermodynamically stable metal oxide was grown (for In and O this is In_2_O_3_. At 500 K, the Gibbs free energy change for the formation of In_2_O is −103.603 kJ/mole, while that of In_2_O_3_ is −765.574 kJ/mole^31^).

**Figure 4:**
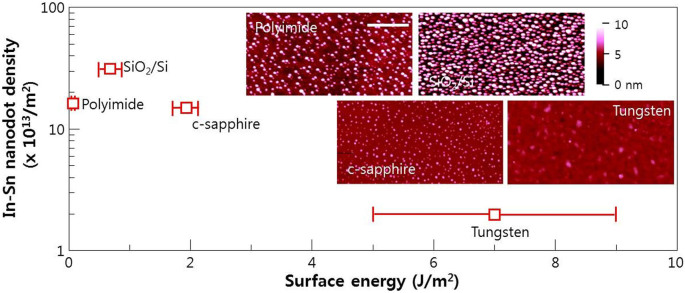
Plot of the In(Sn) nanodot density as a function of the surface energy of the various substrates. The inset shows the AFM images of In(Sn) nanodot. Scale bar, 200 nm.

In(Sn) nanodots were grown at 300°C for 6 s, and observed using atomic force microscopy (AFM); their density was plotted (Fig. 4) as a function of the surface energy of various substrates (Supplementary table S2)^32^,^33^,^34^,^35^. Because the metallic nanodot density may be strongly related to the wetting and spreading characteristics, we considered the contact angle *θ_old_* based on Young's relation: *σ_sl_ − σ_sv_ + σ_lv_* cos *θ_old_* = 0. This describes the conditions for the stability of a liquid droplet on a flat substrate in coexistence with its vapor, in terms of the relevant surface energies *σ_lv_* (liquid/vapor), *σ_sv_* (substrate/vapor) and *σ_sl_* (substrate/liquid). The *σ_lv_* surface energy of liquid In is ~0.6 J/m^2^^36^. Consequently, if the interfacial energies between liquid In and the substrates used in our AFM studies can be obtained, the *θ_old_* values can be calculated. We chose four substrates with different *σ_sv_*: W, Sapphire, SiO_2_, and polyimide (supplementary table S2).

**Figure 5:**
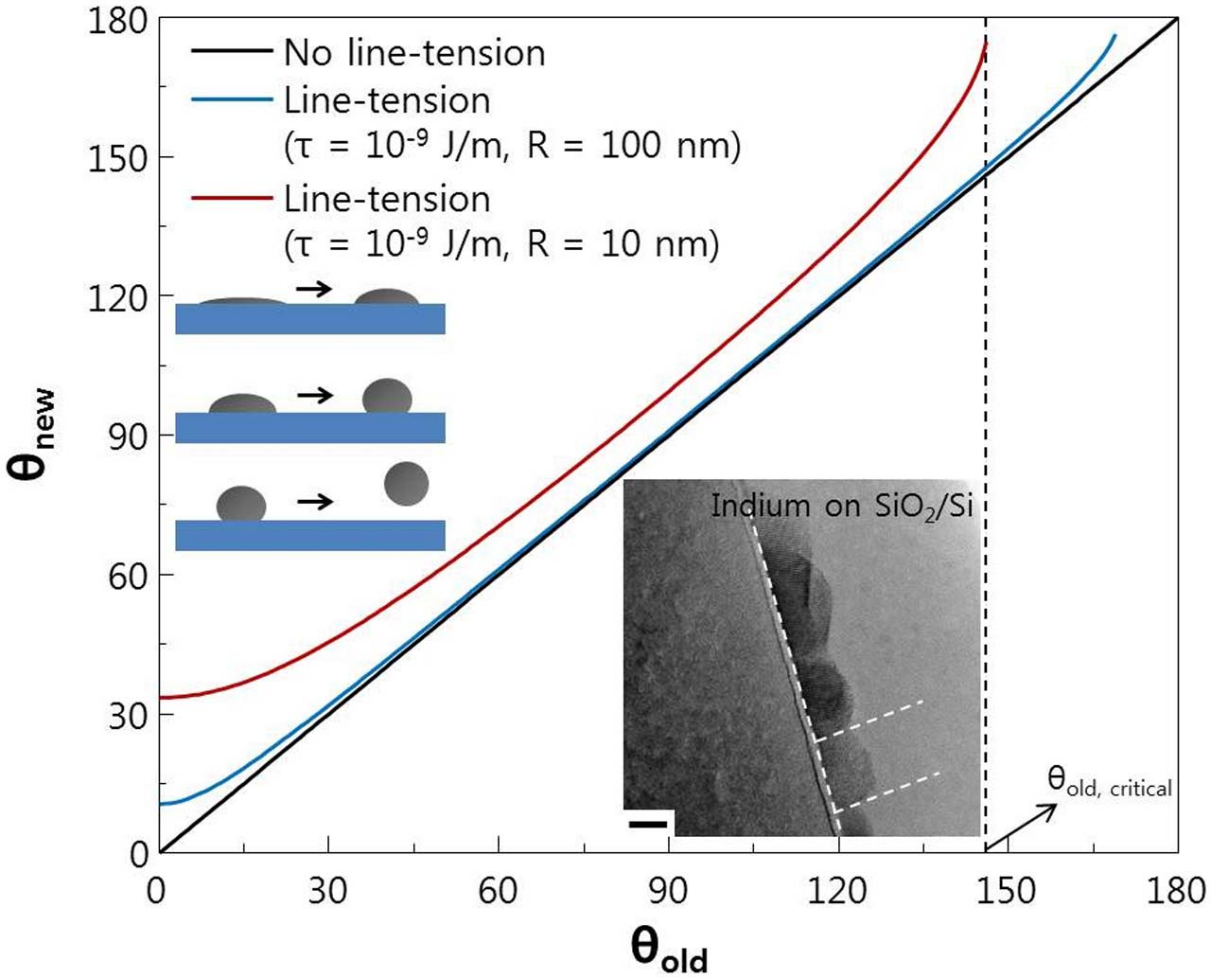
Line tension effect with respect to the diameter of liquid metal (for the case of 10 nm and 100 nm radius). The line tension value was set as 10^−9^ J/m. The inset is TEM image of indium nanoparticle on SiO_2_/Si substrate. The contact angle between SiO_2_ and indium was about 90°. Scale bar, 10 nm.

- W surface: As shown in the reference study of Sb-doped Cu droplet on W surfaces^37^, the surface energy of liquid Cu can be controlled to be between 0.5 J/m^2^ and 1.3 J/m^2^. The surface energy of 0.6 J/m^2^ could also be acquired by doping Sb at about a 15 ~ 20% molar fraction in Cu, which is the same value as for pure indium. In this case, the interfacial energy between the Sb-doped Cu and W is about 6.5 J/m^2^ if we assume the surface energy of solid W (*σ_sv_*) to be about 7 J/m^2^. Accordingly, the contact angle was calculated to be about 30°. Because W has very high surface energy compared to other materials, the metal on this surface tends to spread easily with a low contact angle. This results in formation of a low density of nanodots (Fig. 4).
- Sapphire surface: As shown in the reference study of liquid metals on a sapphire system^38^, the interfacial energy between sapphire and liquid metal at just above the melting point of the metal is about 2.3 J/m^2^. So, if we assume the surface energy of solid sapphire (*σ_sv_*) to be about 1.9 J/m^2^, the contact angle of liquid indium can be calculated to be approximately as about 130°. This value is higher than that calculated for In on a W surface, and results in a relative increase of the density of nanodots at the early stages of growth (without the wetting that occurs on a W surface).
- SiO_2_ surface: As shown in the reference study^39^, the interfacial energy between SiO_2_ and liquid indium is about 0.68 J/m^2^. If we then assume the surface energy solid SiO_2_ (*σ_sv_*) is to be about 0.8 J/m^2^, the contact angle of liquid indium can be calculated to be approximately 80°. The increase of nanodot density on the SiO_2_ surface compared to the sapphire surface can be explained by introducing the line tension effect (Fig. 5).
- Polyimide surface: The only reference study available seems to be that of a Cu droplet on polyimide. In this study^40^, the interfacial energy between Cu and polyimide was calculated to be about 1.6 J/m^2^. Normally, the surface energy of a solid polyimide surface (*σ_sv_*) is below 0.05 J/m^2^. In this case, cos *θ_old_* ≪ −1, so *θ_old_* is out of range. The polymer film prepared by spin coating and curing can have different surface properties compared to the metal and oxide surfaces studied herein. As a result, we cannot estimate the exact contact angle of liquid indium on polyimide.

Next, we considered the effect of line tension effect on *θ_old_* (Fig. 5). As droplet size decreases below the nanometer range, the line tension (*τ*: three phase contact line) effect should be considered. Therefore, the new contact angle should be rewritten as: cos *θ_new_* = cos *θ_old_ − τ*/(*σ_lv_R*), where *R* is the radius of the solid−liquid contact area^41^,^42^,^43^. In a vacuum, metal nanoparticles normally have *θ_new_* ≈ 10^−9^ N/m^44^. The radius of an In nanodot is ~10 nm (inset, Fig. 5). Using this information, we can draw a relation between *θ_old_* and *θ_new_*. The new contact angle equation implies that *θ_new_* > *θ_old_*. The critical contact angle of *θ_old_* (where *θ_new_* = 180°) is ~146°. *θ_new_* of the In nanodot on SiO_2_ is ~90° (*θ_old_* = 80°), which is similar to the value shown in the inset TEM image of Fig. 5. Moreover, the decrease of the density of the In nanodots on a sapphire surface compared to an SiO_2_ surface could be understood to cause the higher *θ_new_* > 145° due to the line tension effect (*θ_old_* ≈ 130°, which is near the critical angle for desorption, which means that *θ_new_* = 180°). The decrease in In(Sn) nanodot density on materials with extremely low surface energy, such as polyimide, could also be understood in the context of the positive line tension effect (desorption from the surface due to high contact angle). The detailed information for the contact angle of In particles on several surfaces are summarized in table 1.

**Figure 6:**
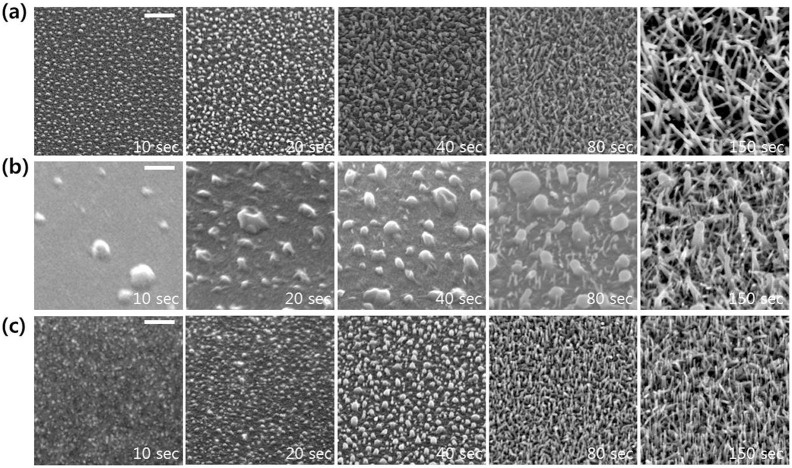
SEM morphology evolution of In(Sn) oxide nanostructures with respect to growth time (a) on SiO_2_/Si substrate, (b) on W coated c-plane sapphire, and (c) on bare c-sapphire substrate. Scale bar, 200 nm.

The perfect wetting and abnormal In(Sn) nanodot formation on the W surface (Fig. 6b) clearly caused difference in the nanostructure development from that on an SiO_2_ surface, which showed uniform nanostructure growth (Fig. 6a). A metal catalyst coated on W surface will change the surface energy of W and tends to cause formation of a large catalyst by a ripening process^45^, resulting in abnormal diameter distribution of ITO nanowires. Moreover, the ITO nanowires on c-plane sapphire (Fig. 6c) had much smaller diameter (~10 nm) than those on the other substrates. This phenomenon can be explained by the high contact angle of liquid In (*θ_old_* ~ 130°; *θ_new_* ~ 145°) on c-sapphire. Increasing contact angle decreases the contact area with the substrate, and therefore decreases the diameter of the ITO nanowires during segregation. ITO nanowires have a larger diameter (>40 nm) on a W surface than on a sapphire surface because liquid In has a lower contact angle (*θ_old_* ~ 30°) on W than on sapphire. If we assume the total volume of metal particles to be constant, we can calculate the contact radius of this particle on several substrates (table 2). This calculation shows a similar tendency to that shown in the SEM image (Fig. 6). To summarize, the surface energy of the substrate effects the contact angle of the liquid droplet at the early stage of growth (from the decomposition of metal-oxide source during electron-beam irradiation), and this effect results in variation of the density and diameter of the metal-oxide nanowires formed.

**Figure 7:**
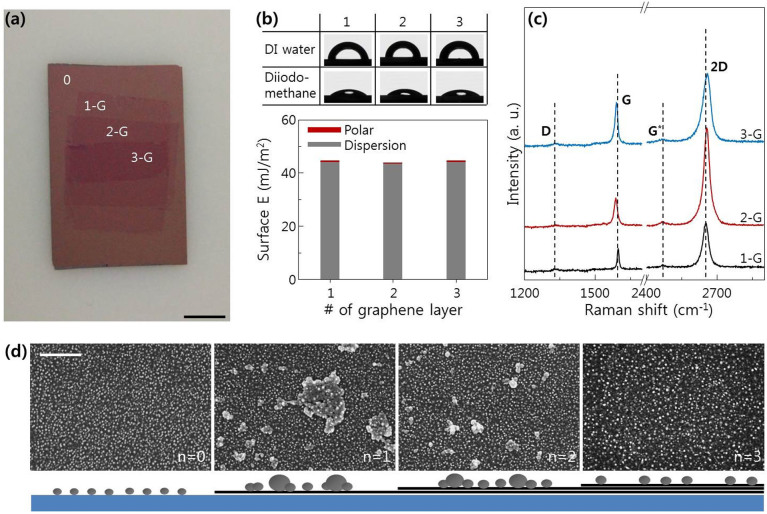
(a) Camera image of graphene transferred 3 times onto SiO_2_/Si(100) surfaces. Scale bar, 1 cm. (b) Contact angle of n-layer graphene using DI water and diiodomethane, and calculated surface energy based on the geometric mean equation. (c) Raman spectrum of n-layer graphene. (d) Top view SEM images of In(Sn) nanodots on n-layer graphene at the early stage of growth and their schematics. The n = 0 layer is bare SiO_2_/Si substrate. Scale bar, 500 nm.

The distribution of In(Sn) nanodot size on the polyimide surface (inset AFM image, Fig. 4) is non-uniform compared to that on SiO_2_ and c-plane sapphire surfaces. To understand this phenomenon, we must consider the surface migration and agglomeration of In(Sn) nanodots. Because polyimide has very low surface energy, the metal nanoparticles on the surface could migrate easily during growth. We compared the distribution of In(Sn) nanodots on specific substrates that had similar surface energy but different surface migration energy, e.g., n-graphene substrate (Fig. 7a)^22^,^23^,^24^. The surface energy of n-graphene is independent of n (Fig. 7b). The surface energy distribution was determined from the contact angle using DI water and diiodomethane as the probe liquids, and calculated using the geometric mean equation: (1 + cosθ)*γ_pl_* = 2(*γ_s_^d^γ_pl_^d^*)^1/2^ + 2(*γ_s_^p^γ_pl_^p^*)^1/2^, where *γ_s_* and *γ_pl_* are the surface energies of the sample and the probe liquid, respectively, and the superscripts *d* and *p* refer to the dispersion and polar (non-dispersion) components of the surface energy, respectively^46^. The calculated surface energy of graphene (~46 to 47 mJ/m^2^) matched quite well with previous reports^47^. However, the surface migration energy of the graphene surface is strongly dependent on n, due to Van der Waals forces between the single graphene layers (Bernal AB stacked graphenes)^22^,^23^,^24^. Although the n-graphenes made by the multiple transfer process do not have perfect AB Bernal stacking order, AB stacking structures can occur over small areas due to the polycrystalline characteristics of CVD graphenes. The Raman spectrum of 1-layer graphene (Fig. 7c) shows the features of monolayer graphene: (i) a 2.0 ~ 3.0 2D/G intensity ratio and (ii) a symmetric 2D band^48^. All of the samples had weak D and G* bands, which is unavoidable in graphene samples grown on polycrystalline copper foil using CVD. The red-shift of the G band and unsymmetrical 2D peaks in the Raman spectrum for 2 and 3-layer graphenes indicated that the anharmonic scattering of optical phonons that are active in the Raman scattering processes increased due to the additional AB stacking graphene layers^49^. When grown at 300°C for 6 s, initial In(Sn) nanodot distribution was affected by the number of graphene layers, from 0 (bare SiO_2_ covered Si) to 3 (Fig. 7d). The strong migration tendency of single-layer graphene caused abnormal nanodot agglomeration. This migration tendency gradually disappeared as the number of graphene layers increased from 1 to 3, (schematics, Fig. 7d) due to strong Van der Waals forces between layers.

Considering all the experimental results, we believe that metal oxides could easily be decomposed by a high density electron-beam, and that sufficient metal flux for the self-catalysis of the VLS process can be formed under a low oxygen partial pressure. Therefore, the metal oxide nanostructures synthesized by the VLS process can be achieved easily at a temperature just above the melting point of the metallic self-catalyst. In this synthesis process, the surface energy and surface migration energy of the substrate strongly effects the density of the self-catalyst distribution, and the final morphology of the nanostructures. The density of the self-catalytic nanodots increased with decreasing surface energy of the substrate, due to the perfect wetting of catalytic materials on high surface energy substrates. However, surfaces with extremely low surface energy have difficulty producing self-catalytic nanodots with high density, due to positive line tension which increases the contact angle > 180°. Moreover, substrates with low surface migration energy, such as single layer graphene, cause the nanodots to agglomerate, thereby producing a non-uniform distribution compared to substrates with high surface migration energy.

## Conclusion

We studied the growth mechanism of metal oxide nanostructures by the simple electron beam evaporation. The high density of the electron beam can easily decompose metal oxides that have high melting points. The decomposed metal elements form the self-catalysts for the VLS process, and the metal oxide nanostructure can be grown at a temperature just above the melting point of the metal. The morphology of the nanostructures, such as density and uniformity, is strongly dependent on the surface energy and surface migration energy of the substrate. Due to its simplicity and scalability, this method to fabricate metal oxide nanostructures could have applications in many fields, such as energy and environmental science, electronics, photonics, and biology.

## Methods

### Electron beam evaporation

The group 13 metal oxide nanostructures (Al_2_O_3_, Ga_2_O_3_, and Sn 10% doped In_2_O_3_) were fabricated by the electron beam evaporation method using high purity (99.99%) oxide pellets as the source materials^50^,^51^. The nanostructures were grown at a rate of 0.5 nm s^−1^, and the chamber pressure was maintained at approximately 10^−5^ Torr during deposition. The substrate temperature was controlled using a heating element made by SiC and calibrated by k-type thermocouple. **Substrate preparation**: Several substrates with different surface energies were prepared; Si(100) covered by thermal oxide at about 200 nm, c-plane sapphire, tungsten film (50 nm) coated by magnetron sputtering (3 mTorr argon plasma, 100 Watt RF power, 300°C) on c-plane sapphire, and spin-coated polyimide film (cured at 150°C) on soda-lime glass. Substrates were prepared and cleaned sequentially with acetone, ethyl-alcohol, and de-ionized water. For the n-layer graphene substrates, the conventional chemical vapor deposition (CVD) method using copper foil as a catalyst was used^52^,^53^. The copper foils (Alfa Aesar, item No. 13382, 99.8% purity) were loaded into the quartz tube reaction chamber. A typical growth process was conducted as follows: (1) the pressure in the growth chamber was pumped down to 5 mTorr using a mechanical pump; (2) a 40 sccm flow of hydrogen gas was introduced into the chamber at 950 mTorr; (3) a the copper foils were heated to 1000°C for 60 min and annealed for 30 min to enlarge the copper grains and remove residual copper oxide and impurities; (4) a 6 sccm flow of methane gas with 20 sccm hydrogen was introduced into the chamber for 10 min with a total pressure of 420 mTorr for graphene synthesis; (5) after growth, the furnace was cooled rapidly to RT under a 20 sccm flow of hydrogen. The synthesize graphenes were transferred onto SiO_2_/Si(100) substrate 3 times to make n-layer graphene surfaces. First, one side of the graphene/copper foils were spin coated with polymethyl methacrylate (PMMA) and dried in atmosphere. Then, the uncoated graphenes were etched with oxygen plasma for 30 sec at 100 W. After the copper foils were totally etched in (NH_4_)_2_S_2_O_8_ solution (0.1 M) for 12 hours, the graphene/PMMA films were washed in DI water several times. The graphene/PMMA films were transferred onto SiO_2_/Si(100) substrate and dried at ambient temperature, then heated at 180°C for 30 min. Finally, the PMMA layers were removed with acetone. **Analysis**: Scanning electron microscopy (SEM) with a PHILIPS XL30S was carried out at an acceleration voltage of 10 kV and working distance of 5 mm. For the elemental analysis at the initial stage of growth, the electron energy loss spectroscopy (EELS) map attached function in high-resolution transmission electron microscopy (HRTEM: model Cs-corrected JEM2200FS operated at 200 kV) was carried out. Atomic force microscopy (AFM) images were recorded using a Digital Instruments Nanoscope in tapping mode with silicon cantilevers. The Raman spectrum was obtained with a Raman spectrometer LabRAM HR 800 from the company HORIBA Yvon GmbH under the following conditions: excitation wavelength of the laser: He-Ne 633 nm; spot size of the laser beam: 5 μm in diameter; measurement time: 10 sec.

## Supplementary Material

Supplementary Information Supplementary information for publication

## Figures and Tables

**Figure 1:**
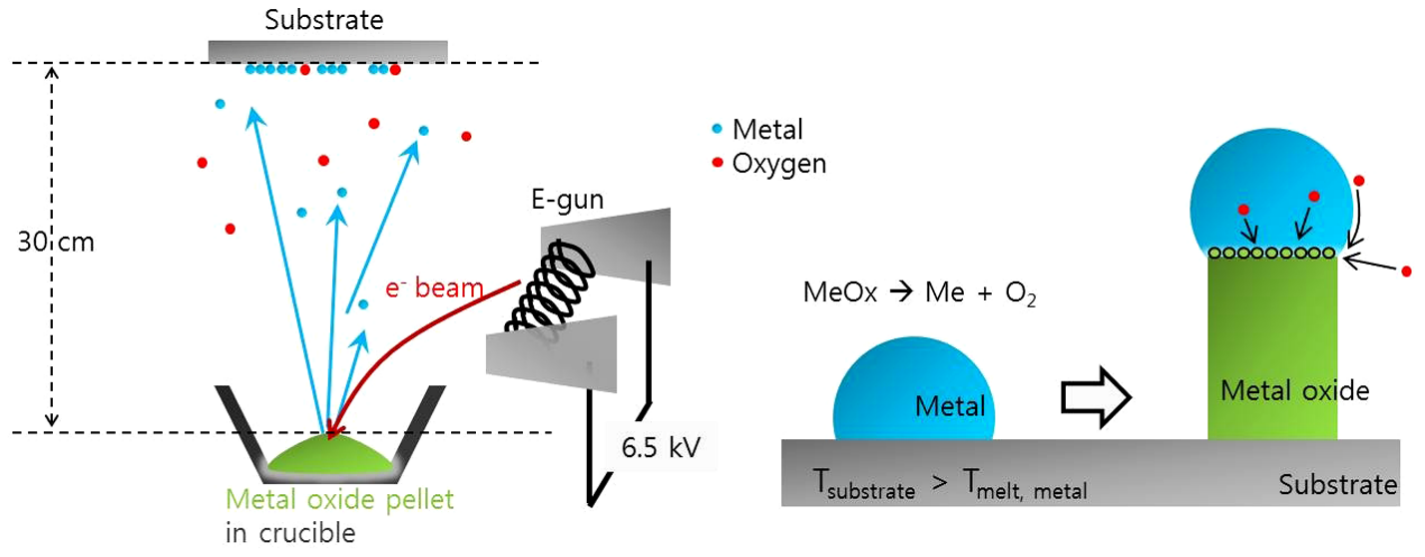
Schematics of the electron beam evaporation model for metal oxide nanowire growth, and growth mechanism by VLS.

**Table 1:** Summarized information for the contact angle of indium particles on several surfaces. The surface energy of liquid indium (σ_lv_) was set as 0.6 J/m^2^ and the line tension of metal nanoparticles in a vacuum (τ) was set as 10^−9^ N/m. The radius of indium nanoparticles (R) was assumed as ~10 nm

	Surface E σ_sv_ (J/m^2^)	Interfacial E with liquid Indium σ_sl_ (J/m^2^)	Calculated θ_old _by cos^−1^ {(σ_sv _− σ_sl_)/σ_lv_}	Calculated θ_new _by cos^−1^ {cos θ_old _− τ/(σ_lv_R)}	Measured θ by TEM
SiO_2_	~0.8	~0.68	80°	90°	90°
C-Al_2_O_3_	~1.9	~2.3	130°	145°	-
W	~7	~6.5	30°	45°	-

**Table 2:** Calculation of contact radius of indium particles on several surfaces. Assume the total volume of liquid indium is same with (4πR_0_^3^)/3, where R_0_ is radius of homogeneous nanoparticle without contact to substrate

	SiO_2_	C-Al_2_O_3_	W
Calculated θ_new_	90°	145°	45°
Contact with Indium NP			
Calculated Contact Radius		*R_sap_* = 0.58 *R*_0_	*R_W_* = 1.82 *R*_0_
