# Supplementary material for: Growth mechanism of metal-oxide nanowires synthesized by electron beam evaporation: A self-catalytic vapor-liquid-solid process
Source: Sci Rep. 2014 Oct 10;4:6589. doi: 10.1038/srep06589 (PMC5377458; doi:10.1038/srep06589)

Supplementary information

Growth mechanism of metal-oxide nanowires synthesized by electron beam evaporation: A self-catalytic vapor-liquid-solid process

Hak Ki Yu[†] and Jong-Lam Lee*

Division of Advanced Materials Science and Department of Materials Science and Engineering, Pohang University of Science and Technology (POSTECH), Pohang, 790-784, Korea.

[†]Present Addresses: Max-Planck-Institut Für Biophysikalische Chemie, am Fassberg 11, 37077 Göttingen, Germany

Corresponding author: jllee@postech.ac.kr

Figure S1. MgO nanowire growth by electron-beam evaporation. The MgO (with very high melting point more than 2800 ^oC, wide band-gap about 7.8 eV, superior breakdown field about 12 mV/cm, small lattice mismatch with functional oxide such as multi-ferroelectrics and superconductors, and good protective sheath in aggressive environments) nanowires can be grown only from the chemical solution route or oxidation of Mg vapor during growth.[Ref 1)] In other words, it is not so easy to use the source material of MgO itself to make nanostructure. Although one group successfully probed the MgO nanowire growth from the oxide source itself by using electron beam attached in TEM, they used the Au nano-catalyst.[Ref 2)] However, we can successfully grow the high density of MgO nanowires from MgO source without any heterogeneous catalyst. So, the electron beam would be interesting method for many other metal-oxide nanowires growth in large area without any contamination. (**a**), (**b**) SEM images of MgO nanowire. MgO nanowires were grown from the MgO pellets made by compressing and heating 99.995% purity MgO powder (Mitsubishi Materials Co.). Chamber pressure was maintained at ~10^{-5} Torr during deposition and the distance from the substrate (SiO_2/Si) and MgO pellet source was quite close about 3 cm. (**c**) HR-TEM image of MgO nanowire. The inset is diffraction pattern of it.

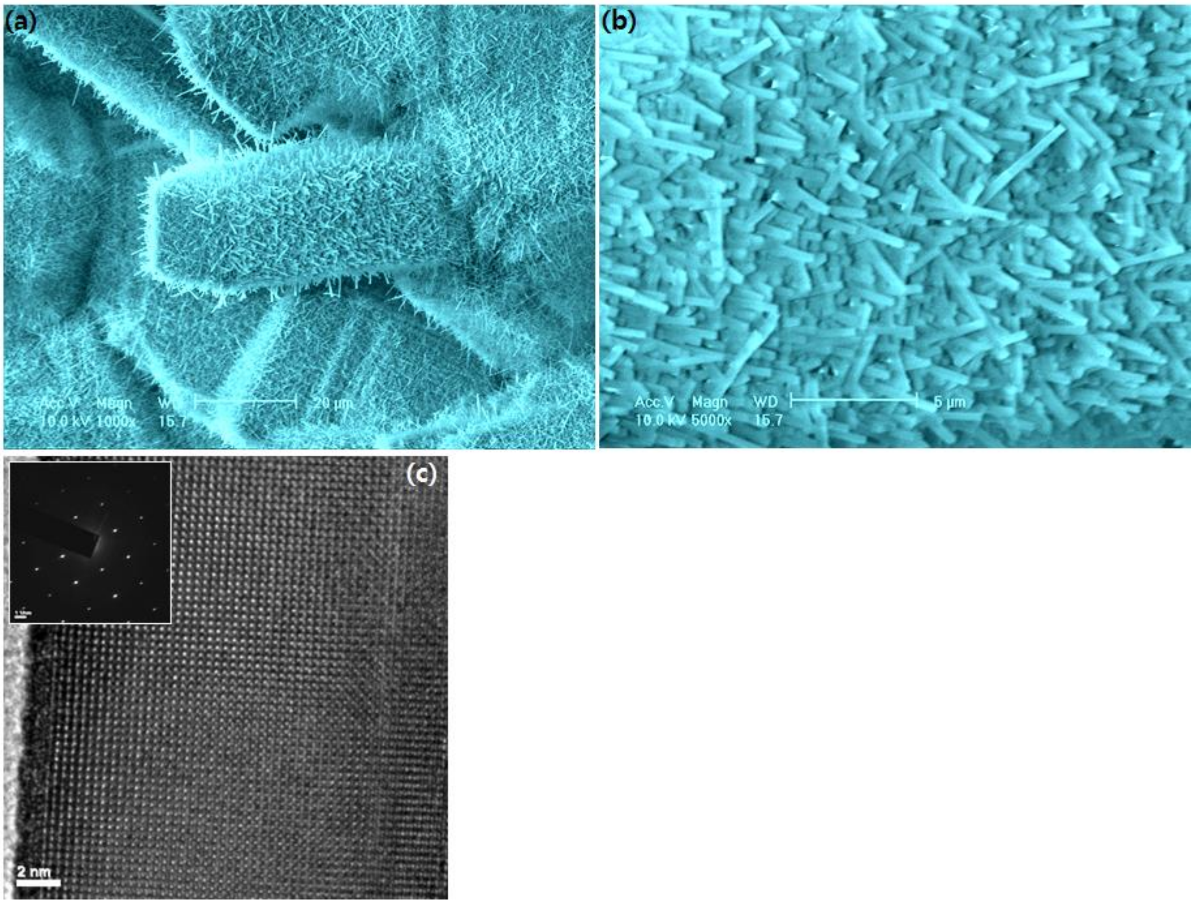

Ref 1) Wie, Q. & Lieber, C. M. Solution based synthesis of magnesium oxide nanorods. *Mater. Res. Soc. Symp. Proc.* **581**, 1-7 (2000).
Ref 2) Nasibulin, A. G. *et al.* In Situ TEM observation of MgO nanorod growth. *Cryst. Growth Des.* **10**, 414-417 (2010).

Table S2. Surface of several materials.

Surface E	Materials
Low surface E<br>(< 0.5 J/m^2)	Polymer and plastic based film (PDMS, PMMA, **Polyimide**, PET, etc)<br>Rubber<br>Carbon based materials (Graphite and **Graphene**)
Moderate surface E<br>(0.5 ~ 2 J/m^2)	Most of metals with moderate melting point<br>**Several oxides** (Strongly depend on the orientation due to anisotropic properties)
High surface E<br>(> 2 J/m^2)	Metals with high melting point, such as **W**, Ir, Re, Ta, Pt, Ru, Tc, Mo, etc.

Ref 3) Vitos, L., Ruban, A. V., Skriver, H. L. & Kollár, J. The surface energy of metals. *Surf. Sci.* **411**, 186-202 (1998)
Ref 4) Owens, D. K. Estimation of the surface free energy of polymers. *J. Appl. Polym. Sci.* **13**, 1741-1747 (1969).
Ref 5) Davidge, R. W. Mechanical behavior of ceramic (Cambridge University Press, 1979).

Figure S3. Scanning Auger spectrum for indium nanodot. Scanning Auger spectrum of spot in inset SEM. The atomic ratio between In and Sn was 10:1. The inset is a 45° tilted SEM image of the initial stage of ITO nanowires growth (for 20 sec on SiO_2/Si substrate). Scale bar, 100 nm.

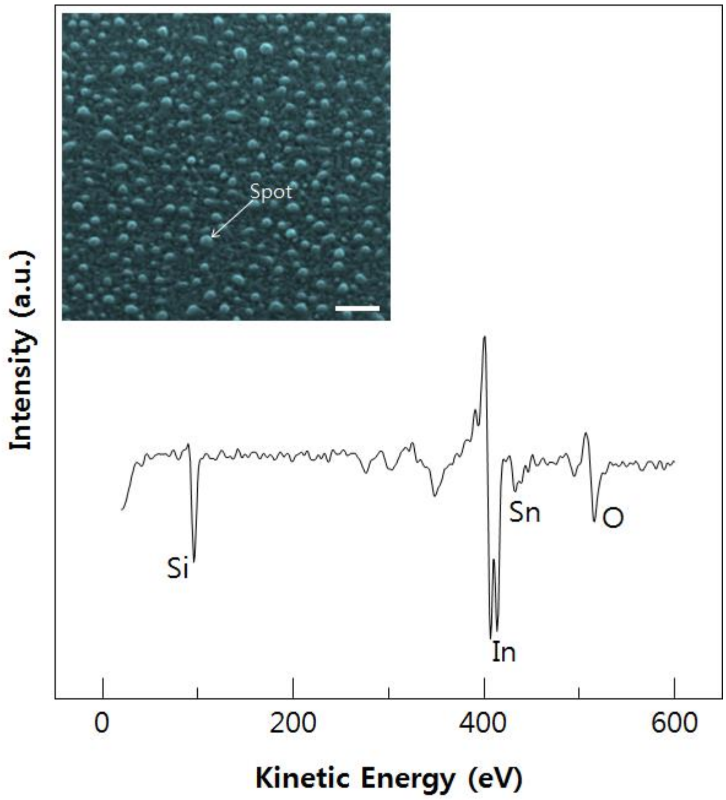

Supplement: Supplementary Information — for publication [file srep06589-s1.pdf]
